# Supplementary material for: Recent community warming of moths in Finland is driven by extinction in the north and colonisation in the south
Source: Nat Commun. 2025 Aug 12;16:7063. doi: 10.1038/s41467-025-62216-9 (PMC12344031; doi:10.1038/s41467-025-62216-9)
Supplement: Supplementary file 1 — Supplementary Information [file 41467_2025_62216_MOESM1_ESM.pdf]

Supplementary Material for:

Recent community warming of moths in Finland is driven by extinction in the north and colonisation in the south.

Ellis E. E.[1*], Antão, L.[1,2], Davrinche, A.[1], Mäkinen, J[1,3], Rees, M.[4], Conenna, I.[1], Huikkonen, I.-M.[3], Leinonen, R. [5], Pöyry, J.[3], Suuronen, A. [3], Laine, A.-L.[1], Saastamoinen, M.[1], Vanhatalo, J.[1,6], and Roslin, T.[1,7]

[1]Research Centre for Ecological Change, Organismal and Evolutionary Research Programme, Faculty of Biological and Environmental Sciences, University of Helsinki, Helsinki, Finland
[2]Department of Biology, Faculty of Science,University of Turku, Finland
[3]Finnish Environment Institute (SYKE), Helsinki, Finland
[4]School of Biosciences, University of Sheffield, U.K.
[5]Kainuu Centre for Economic Development, Transport and the Environment, Kajaani, Finland
[6]Department of Mathematics and Statistics, Faculty of Science, University of Helsinki, Helsinki, Finland
[7]Department of Ecology, Swedish University of Agricultural Sciences (SLU), Uppsala, Sweden

* Corresponding author:
Emilie E. Ellis,
Research Centre for Ecological Change,
Organismal and Evolutionary Research Programme,
Faculty of Biological and Environmental Sciences,
University of Helsinki,
Finland
Emilie.ellis@helsinki.fi // emilie.ellis95@gmail.com

Table of Contents

Supplementary Figures: ... 3
Figure 1: Shown on the left is a map of north-east Europe with Finland (the study system) outlined in blue. Shown on the right are latitudinal patterns in ecological communities and abiotic processes of likely importance to the ability and rate at which communities will undergo thermophilisation. ... 3
Figure 2: Spatial distribution of sampling sites and changes in biotic temperatures over time. A) Map of Finland showing the distribution of sampling sites across three distinct bioclimatic zones, north boreal (blue), mid boreal (green) and south boreal (yellow), B) The community weighted mean of species temperature indexes (CTI) of each site plotted along a thirty-year time series. The points denote site- and year-specific CTI, and the colour identifies the bioclimatic zone of the site. Lines are predictions from linear mixed effect models showing a significant increase in CTI through time, with significant variation in rates of change among sites (i.e. a significant site*year interaction). ... 4
Figure 3: Correlation matrix of mean temperature (meanTemp = species temperature index), and derived monthly growing degree day (GDD) temperatures in February (est_GDD_Feb), April (est_GDD_April), June(est_GDD_June) and August (est_GDD_August). ... 5
Figure 4: The four thermal groups of moths based on the quartile ranges of the species temperature index (STI). The Table shows the range of temperatures in each group, and the figure illustrates the distribution of the data. ... 6
Figure 5: Defining the local species pool. Schematic showing how species pools were defined. A) all species in our dataset were assigned a range, defined by the minimum and maximum latitude of their distribution (depicted by dashed lines). B) The species pool of a site was then defined as all species the ranges of which overlap with the site's location. ... 7
Figure 6: Correlation matrix of our explanatory variables. ... 8

Supplementary Tables: ... 9
Table 1: Regression Analysis Summary testing how community temperature index (CTI) is changing through time (year), and space (latitude) and their interaction. Model fitted as a linear mixed model with site as a random effect ... 9
Table 2: Maximum likelihood of colonisation-extinction rate pairs in each site fitted to site latitude, insect abundance (total insect abundance averaged across years), and available species (species richness of the species that could colonise at site). Two-sided Wald z-tests were used for maximum likelihood parameter estimation (using the bbmle R package). ... 10
Table 3: Maximum likelihood of colonisation-extinction rate pairs for four thermal groups in each site fitted to site latitude, insect abundance (total insect abundance averaged across years), and available species (species richness of the species that could colonise at site averaged across years). Two-sided Wald z-tests were used for maximum likelihood parameter estimation (using the bbmle R package). ... 11
Table 4: Sample coverage estimates for 62 sites in Finland, showing the reference sample size (number of observations), the species richness (observed) and the sample coverage (estimated from rarefaction and 1000 iterations. F1-F5 are the first ten frequency counts. ... 14
Table 5: Maximum likelihood of colonisation-extinction rate pairs in each site fitted to site latitude, insect abundance (total insect abundance averaged across years), and available species (species richness of the species that could colonise at site) with singleton species removed. Two-sided Wald z-tests were used for maximum likelihood parameter estimation (using the bbmle R package). ... 16
Table 6: Maximum likelihood of colonisation-extinction rate pairs for four thermal groups in each site fitted to site latitude, insect abundance (total insect abundance averaged across years), and available species (species richness of the species that could colonise at site averaged across years) with singleton species removed. Two-sided Wald z-tests were used for maximum likelihood parameter estimation (using the bbmle R package). ... 17
Table 7: R packages used (including the version and citation): ... 20
Table 8: Moth families included as 'macromoths' ... 21

Supplementary Figures:

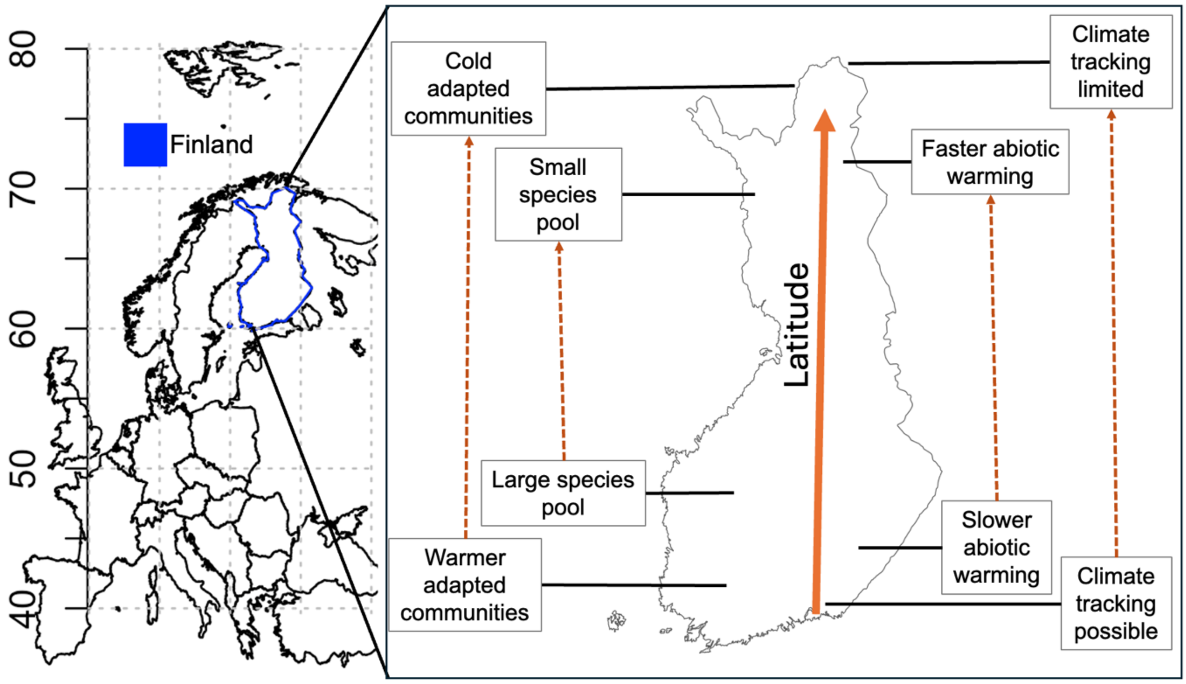

Figure 1: Shown on the left is a map of north-east Europe with Finland (the study system) outlined in blue. Shown on the right are latitudinal patterns in ecological communities and abiotic processes of likely importance to the ability and rate at which communities will undergo thermophilisation.

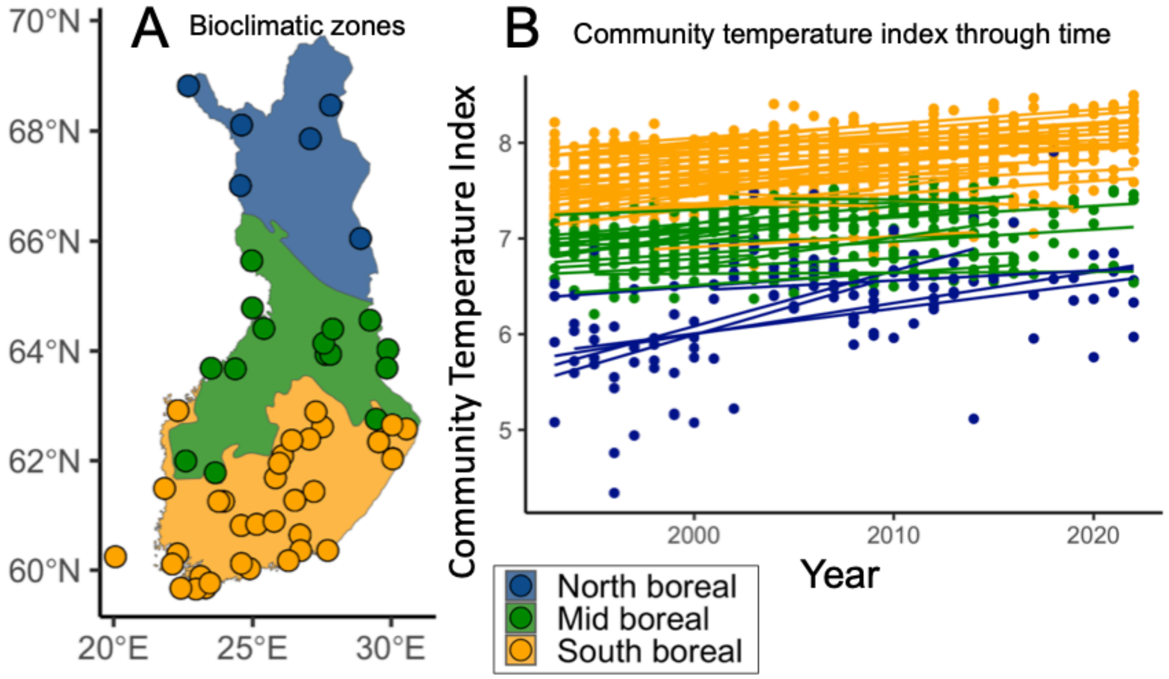

Figure 2: Spatial distribution of sampling sites and changes in biotic temperatures over time. A) Map of Finland showing the distribution of sampling sites across three distinct bioclimatic zones, north boreal (blue), mid boreal (green) and south boreal (yellow), B) The community weighted mean of species temperature indexes (CTI) of each site plotted along a thirty-year time series. The points denote site- and year-specific CTI, and the colour identifies the bioclimatic zone of the site. Lines are predictions from linear mixed effect models showing a significant increase in CTI through time, with significant variation in rates of change among sites (i.e. a significant site*year interaction).

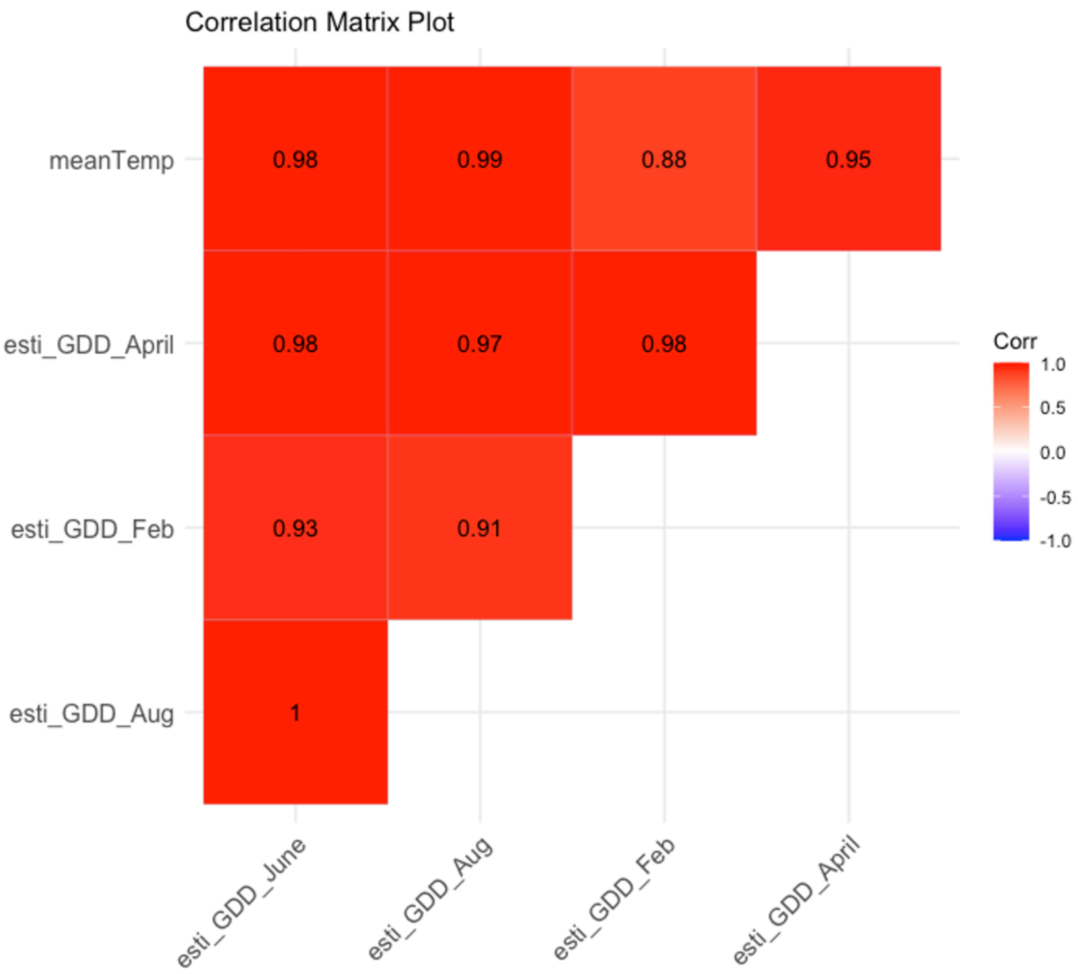

Figure 3: Correlation matrix of mean temperature (meanTemp = species temperature index), and derived monthly growing degree day (GDD) temperatures in February (est_GDD_Feb), April (est_GDD_April), June(est_GDD_June) and August (est_GDD_August).

Quartile	Range (°C)	Grouping
25%	-0.56 – 7.29	Cold adapted
50%	7.291 – 8.23	Cold tolerant
75%	8.231 – 8.86	Warm tolerant
100%	8.861 – 13.44	Warm adapted

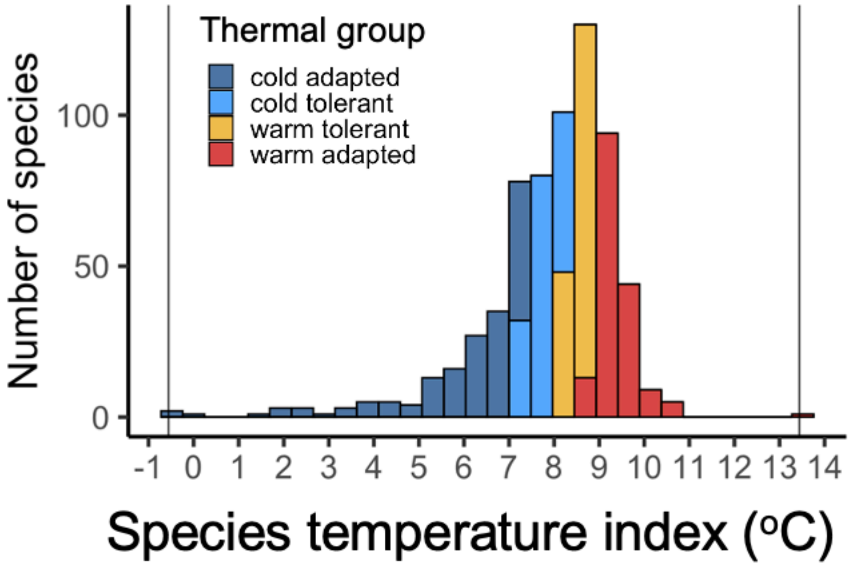

Figure 4: The four thermal groups of moths based on the quartile ranges of the species temperature index (STI). The Table shows the range of temperatures in each group, and the figure illustrates the distribution of the data.

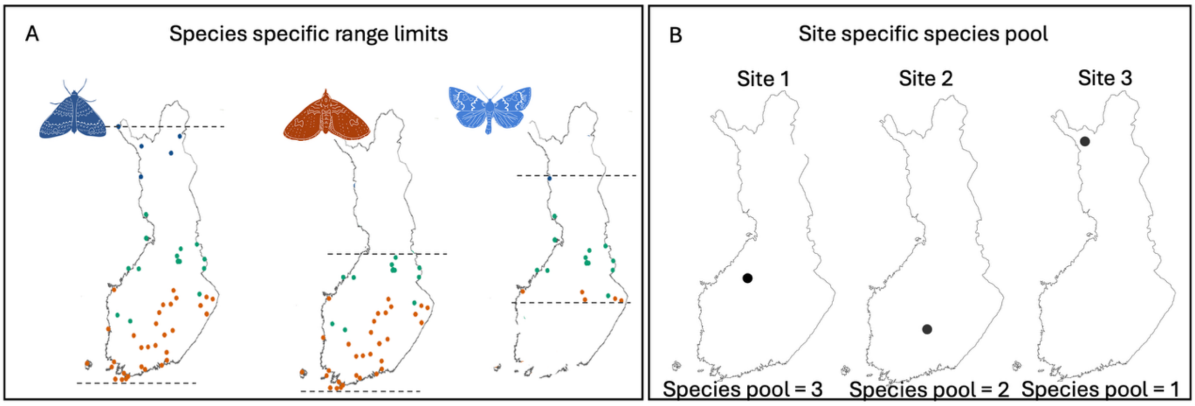

Figure 5: Defining the local species pool. Schematic showing how species pools were defined. A) all species in our dataset were assigned a range, defined by the minimum and maximum latitude of their distribution (depicted by dashed lines). B) The species pool of a site was then defined as all species the ranges of which overlap with the site's location.

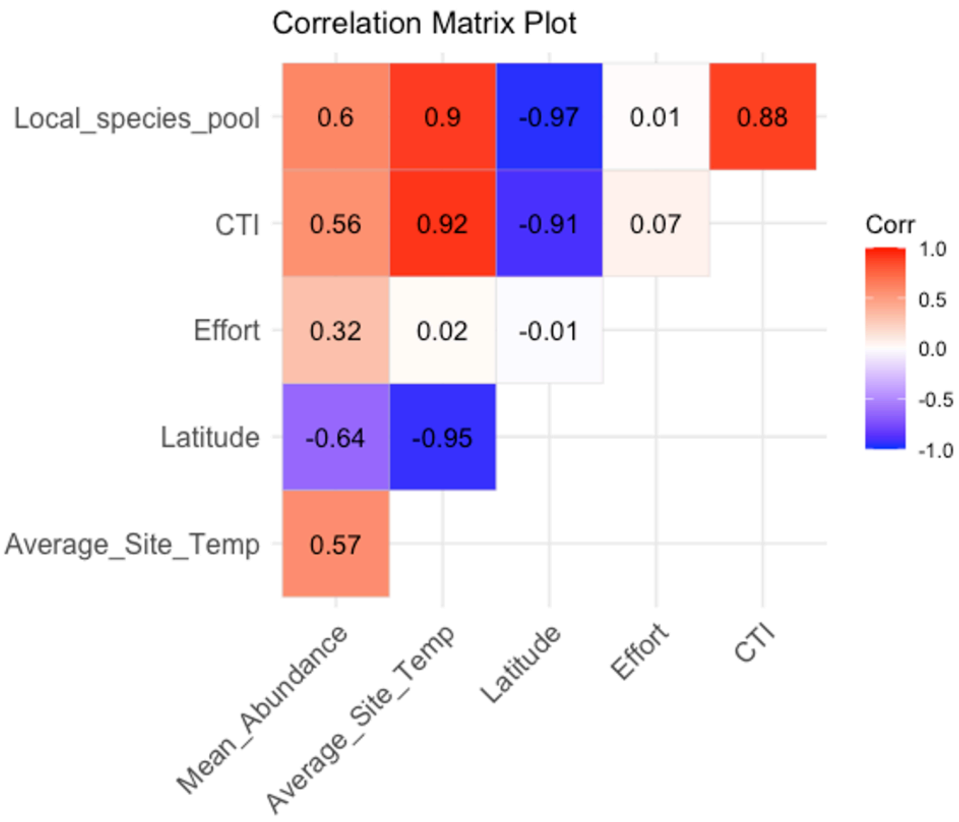

Figure 6: Correlation matrix of our explanatory variables.

Supplementary Tables:

Table 1: Regression Analysis Summary testing how community temperature index (CTI) is changing through time (year), and space (latitude) and their interaction. Model fitted as a linear mixed model with site as a random effect

CTI ~ year*lat+(1|Site)			
Coefficent	Estimate	Error	t value
(Intercept)	246.60	40.79	6.05
as.numeric(Year)	-0.11	0.02	-5.56
latitude	-42.71	6.53	-6.54
as.numeric(Year):latitude	0.02	0.00	6.24

Table 2: Maximum likelihood of colonisation-extinction rate pairs in each site fitted to site latitude, insect abundance (total insect abundance averaged across years), and available species (species richness of the species that could colonise at site). Two-sided Wald z-tests were used for maximum likelihood parameter estimation (using the bbmle R package).

Response variable	Coefficients:				
**Latitude**		Estimate	Std. Error	z value	p-value
	Extinction intercept	-6.46	0.16	-40.31	< 2.2e-16
	Extinction slope	0.85	0.03	33.05	< 2.2e-16
	Colonisation intercept	1.13	0.16	7.02	2.202e-12
	Colonisation slope	-0.37	0.03	-14.24	< 2.2e-16
Likelihood (-2log)	463322.1				
**Insect abundance**					
	Extinction intercept	2.41	0.06	36.50	< 2.2e-16
	Extinction slope	-0.32	0.01	-54.12	< 2.2e-16
	Colonisation intercept	-0.99	0.07	-14.79	< 2.2e-16
	Colonisation slope	0.03	0.007	4.423	9.699e-06
Likelihood (-2log)	461927				
**Available species pool**					
	Extinction intercept	0.6144	0.10	17.41	< 2.2e-16
	Extinction slope	-0.30	0.02	-28.82	< 2.2e-16
	Colonisation intercept	-1.98	0.11	-21.81	< 2.2e-16
	Colonisation slope	0.13	0.02	11.64	1.12e-15
Likelihood (-2log)	463881.6				

Table 3: Maximum likelihood of colonisation-extinction rate pairs for four thermal groups in each site fitted to site latitude, insect abundance (total insect abundance averaged across years), and available species (species richness of the species that could colonise at site averaged across years). Two-sided Wald z-tests were used for maximum likelihood parameter estimation (using the bbmle R package).

Model	Explanitory varible	Group	Coefficents	Estimate	Error	z-score	p-value
**i**	**Latitude**						
		Cold adapted					
			Extinction intercept	-5.17	0.27	-19.33	**< 2.2e-16**
			Extinction slope	0.63	0.04	14.72	**< 2.2e-16**
			Colonisation intercept	-0.73	0.25	-2.93	**1.428e-13**
			Colonisation slope	-0.06	0.04	-1.48	0.1386
			Negative Log Likelihood				141666.7
		Cold tolerant					
			Extinction intercept	-7.64	0.30	-25.48	**<2.2e-16**
			Extinction slope	1.05	0.05	21.67	**<2.2e-16**
			Colonisation intercept	2.53	0.30	8.32	**<2.2e-16**
			Colonisation slope	-0.58	0.05	-11.92	**<2.2e-16**
			Negative Log Likelihood				138223.8
		Warm tolerant					
			Extinction intercept	-10.67	0.36	-29.25	**<2.2e-16**
			Extinction slope	1.54	0.03	26.01	**<2.2e-16**
			Colonisation intercept	4.50	0.37	12.28	**<2.2e-16**
			Colonisation slope	-0.91	0.06	-15.37	**<2.2e-16**
			Negative Log Likelihood				120511.7
		Warm adapted					
			Extinction intercept	-13.85	0.56	-24.51	**<2.2e-16**
			Extinction slope	2.12	0.09	22.82	**<2.2e-16**
			Colonisation intercept	5.41	0.63	8.58	**<2.2e-16**
			Colonisation slope	-1.11	0.10	-10.73	**<2.2e-16**
			Negative Log Likelihood				66121.67
**ii**	**Insect abundance**						
		Cold adapted					
			Extinction intercept	1.51	0.12	12.79	**<2.2e-16**
			Extinction slope	-0.27	0.01	-23.24	**<2.2e-16**
			Colonisation intercept	-1.25	0.11	-11.21	**<2.2e-16**
			Colonisation slope	0.02	0.01	1.41	0.16
			Negative Log Likelihood				141297.50
		Cold tolerant					

		Extinction intercept	2.27	0.10	22.95	**<2.2e-16**
		Extinction slope	-0.35	0.01	-34.56	**<2.2e-16**
		Colonisation intercept	-1.43	0.10	-13.65	**<2.2e-16**
		Colonisation slope	0.04	0.01	3.31	0.00095
		Negative Log Likelihood				137622.50
	Warm tolerant					
		Extinction intercept	2.08	0.09	22.57	**<2.2e-16**
		Extinction slope	-0.33	0.01	-35.48	**<2.2e-16**
		Colonisation intercept	-1.78	0.09	-19.19	**<2.2e-16**
		Colonisation slope	0.07	0.01	7.04	**<2.2e-16**
		Negative Log Likelihood				120182.60
	Warm adapted					
		Extinction intercept	1.23	0.09	14.27	**<2.2e-16**
		Extinction slope	-0.25	0.01	-25.57	**<2.2e-16**
		Colonisation intercept	-1.82	0.08	-21.39	**<2.2e-16**
		Colonisation slope	0.05	0.01	5.55	**1.897e-05**
		Negative Log Likelihood				65977.23
**iii**	**Available species pool**					
	Cold adapted					
		Extinction intercept	0.15	0.12	1.26	**0.004269**
		Extinction slope	-0.300	0.02	-11.54	**<2.2e-16**
		Colonisation intercept	-1.87	0.11	-16.26	**<2.2e-16**
		Colonisation slope	0.168	0.024	6.78	**7.613e-16**
		Negative Log Likelihood				141665.5
	Cold tolerant					
		Extinction intercept	0.40	0.095	4.20	**0.7187**
		Extinction slope	-0.33	0.020	-16.44	**<2.2e-16**
		Colonisation intercept	-2.09	0.11	-18.98	**<2.2e-16**
		Colonisation slope	0.21	0.024	9.08	**<2.2e-16**
		Negative Log Likelihood				138603.4
	Warm tolerant					
		Extinction intercept	0.83	0.097	8.57	**1.024e-13**
		Extinction slope	-0.44	0.021	-21.01	**<2.2e-16**
		Colonisation intercept	-2.70	0.115	-23.54	**<2.2e-16**
		Colonisation slope	0.34	0.025	13.62	**<2.2e-16**
		Negative Log Likelihood				120957.4
	Warm adapted					
		Extinction intercept	0.76	0.09	8.68	**<2.2e-16**
		Extinction slope	-0.42	0.021	-19.82	**<2.2e-16**
		Colonisation intercept	-2.22	0.092	-24.17	**<2.2e-16**
		Colonisation slope	0.21	0.02	9.49	**<2.2e-16**

	Negative Log Likelihood	66172.39

Table 4: Sample coverage estimates for 62 sites in Finland, showing the reference sample size (number of observations), the species richness (observed) and the sample coverage (estimated from rarefaction and 1000 iterations. F1-F5 are the first ten frequency counts.

Site	Reference sample size	Species richness	Sample coverage	f1	f2	f3	f4	f5
ID_1001	7187	254	0.9905	68	27	17	11	11
ID_1003	47105	305	0.9991	41	24	15	11	9
ID_102	227737	543	0.9998	40	22	18	13	10
ID_104	206614	480	0.9999	30	11	12	11	11
ID_110	48327	487	0.9990	46	32	26	12	16
ID_1101	21042	254	0.9980	43	27	14	9	9
ID_1102	24042	215	0.9985	37	17	10	7	7
ID_1113	26831	147	0.9989	30	11	7	5	7
ID_1115	33710	270	0.9988	41	21	13	15	7
ID_112	44877	435	0.9988	54	34	21	22	22
ID_114	95294	548	0.9997	33	25	19	14	10
ID_1202	15604	190	0.9976	38	27	9	8	4
ID_1204	75568	251	0.9995	35	20	10	4	12
ID_1206	96169	322	0.9996	34	17	14	19	5
ID_1209	18395	251	0.9976	45	20	12	9	5
ID_1210	36330	250	0.9993	27	19	10	6	9
ID_1212	64252	266	0.9994	40	15	9	11	10
ID_1214	116392	336	0.9997	31	23	17	8	11
ID_1262	109859	246	0.9997	33	23	18	14	4
ID_1304	4865	59	0.9973	13	5	3	4	2
ID_1306	2088	51	0.9938	13	5	4	3	3
ID_1308	22995	61	0.9995	12	8	7	1	2
ID_1310	35469	240	0.9991	33	16	10	10	8
ID_1312	11365	134	0.9982	20	7	16	2	4
ID_1316	2600	66	0.9923	20	2	6	0	4
ID_155	287422	571	0.9999	38	17	16	9	6
ID_158	64419	517	0.9991	57	25	22	13	11
ID_165	178651	515	0.9998	31	23	12	11	11
ID_169	197688	554	0.9998	36	18	15	11	6
ID_201	199149	517	0.9998	41	25	16	13	13
ID_204	89061	473	0.9996	34	26	17	10	17
ID_206	157060	516	0.9997	42	16	14	15	18
ID_210	27770	374	0.9982	49	21	23	14	20
ID_211	39027	463	0.9987	51	29	23	24	14
ID_212	74929	469	0.9993	49	24	25	12	9
ID_303	74692	375	0.9995	41	28	21	15	10
ID_306	35645	323	0.9990	37	16	10	13	13
ID_309	10678	342	0.9944	60	28	23	18	18
ID_310	150925	483	0.9998	31	16	13	9	16

ID_312	38888	386	0.9990	39	31	23	16	7
ID_402	43185	428	0.9990	44	25	27	9	10
ID_403	62990	480	0.9993	43	22	12	17	18
ID_412	65931	510	0.9992	51	29	18	25	12
ID_506	52485	381	0.9992	44	19	18	21	14
ID_507	22871	357	0.9979	48	29	23	14	9
ID_508	46044	364	0.9992	36	22	6	17	11
ID_601	58054	378	0.9993	40	26	13	13	12
ID_603	30598	371	0.9982	54	20	22	15	12
ID_609	36492	332	0.9989	39	27	14	11	9
ID_701	34985	387	0.9985	54	33	22	12	14
ID_702	55626	352	0.9991	48	22	11	9	8
ID_703	56902	388	0.9994	36	17	16	14	15
ID_705	41591	397	0.9989	46	35	14	17	16
ID_706	60662	403	0.9993	45	22	17	16	11
ID_708	31557	279	0.9987	40	20	14	11	8
ID_710	70048	328	0.9994	45	20	17	16	6
ID_802	68341	364	0.9995	32	21	15	12	16
ID_811	43212	294	0.9989	49	34	24	7	8
ID_903	58139	401	0.9992	47	21	11	9	8
ID_908	117454	435	0.9997	35	14	15	7	13
ID_909	87586	400	0.9995	43	24	12	14	9
ID_910	47550	379	0.9989	52	34	12	12	14

Table 5: Maximum likelihood of colonisation-extinction rate pairs in each site fitted to site latitude, insect abundance (total insect abundance averaged across years), and available species (species richness of the species that could colonise at site) with singleton species removed. Two-sided Wald z-tests were used for maximum likelihood parameter estimation (using the bbmle R package).

Response variable	Coefficients:				
**Latitude**		Estimate	Std. Error	z value	p-value
	extinction intercept	-6.355933	0.167498	-37.9463	< 2.2e-16
	extinction slope	0.838068	0.027067	30.9629	< 2.2e-16
	colonisation intercept	0.813698	0.169634	4.7968	1.612e-06
	colonisation slope	-0.286513	0.027415	-10.4511	< 2.2e-16
Likelihood (-2log)	426191.9				
**Insect abundance**					
	extinction intercept	2.3071649	0.0695707	33.1629	< 2.2e-16
	extinction slope	- 0.3110321	0.0062142	-50.0522	< 2.2e-16
	colonisation intercept	- 0.3739220	0.0711469	-5.2556	1.475e-07
	colonisation slope	- 0.0520424	0.0063538	-8.1908	2.595e-16
Likelihood (-2log)	424949.3				
**Available species pool**					
	extinction intercept	1.659956	0.110950	14.9613	< 2.2e-16
	extinction slope	-0.452961	0.017693	-25.6005	< 2.2e-16
	colonisation intercept	-1.789515	0.120145	-14.8946	< 2.2e-16
	colonisation slope	0.132379	0.019172	6.9047	5.03e-12
Likelihood (-2log)	426605.2				

Table 6: Maximum likelihood of colonisation-extinction rate pairs for four thermal groups in each site fitted to site latitude, insect abundance (total insect abundance averaged across years), and available species (species richness of the species that could colonise at site averaged across years) with singleton species removed. Two-sided Wald z-tests were used for maximum likelihood parameter estimation (using the bbmle R package).

Model	Explanitory varible	Group	Coefficents	Estimate	Error	z-score	p-value
i	Latitude						
		Cold adapted	Extinction intercept	-5.082178	0.278011	-18.2805	< 2e-16 ***
			Extinction slope	0.617156	0.044538	13.8568	< 2e-16 ***
			Colonisation intercept	-0.555549	0.262393	-2.1172	0.03424 *
			Colonisation slope	-0.052938	0.042011	-1.2601	0.20763
			Negative Log Likelihood				130925.8
		Cold tolerant					
			Extinction intercept	-7.353618	0.327077	-22.4828	< 2.2e-16 ***
			Extinction slope	1.002498	0.052837	18.9736	< 2.2e-16 ***
			Colonisation intercept	1.951572	0.334139	5.8406	5.202e-09 ***
			Colonisation slope	-0.458217	0.053954	-8.4927	< 2.2e-16 ***
			Negative Log Likelihood				126960
		Warm tolerant					
			Extinction intercept	-10.191725	0.381104	-26.742	< 2.2e-16 ***
			Extinction slope	1.459933	0.061831	23.611	< 2.2e-16 ***
			Colonisation intercept	3.267208	0.381669	8.560	< 2.2e-16 ***
			Colonisation slope	-0.684003	0.061899	-11.050	< 2.2e-16 ***
			Negative Log Likelihood				111694
		Warm adapted					
			Extinction intercept	-12.35101	0.69150	-17.8611	< 2.2e-16 ***
			Extinction slope	1.86542	0.11360	16.4206	< 2.2e-16 ***
			Colonisation intercept	5.00081	0.70632	7.0801	1.441e-12 ***
			Colonisation slope	-1.00892	0.11603	-8.6950	< 2.2e-16 ***
			Negative Log Likelihood				59019.24
ii	Insect abundance						
		Cold adapted					
			Extinction intercept	1.432306	0.123606	11.5877	< 2.2e-16 ***

		Extinction slope	-0.258843	0.012002	-21.5672	< 2.2e-16 ***
		Colonisation intercept	-0.663415	0.116939	-5.6731	1.402e-08 ***
		Colonisation slope	-0.021204	0.011340	-1.8699	0.0615 .
		Negative Log Likelihood				130659.6
	Cold tolerant					
		Extinction intercept	2.3305144	0.1089350	21.3936	< 2.2e-16 ***
		Extinction slope	-0.3527365	0.0110394	-31.9524	< 2.2e-16 ***
		Colonisation intercept	-0.8638349	0.1155275	-7.4773	7.586e-14 ***
		Colonisation slope	-0.0016005	0.0117182	-0.1366	0.8914
		Negative Log Likelihood				126404.7
	Warm tolerant					
		Extinction intercept	1.9266732	0.0963310	20.0006	< 2.2e-16 ***
		Extinction slope	-0.3173176	0.0097633	-32.5010	< 2.2e-16 ***
		Colonisation intercept	-1.2076357	0.0987945	-12.2237	< 2.2e-16 ***
		Colonisation slope	0.0264905	0.0100274	2.6418	0.008246 **
		Negative Log Likelihood				111393.6
	Warm adapted					
		Extinction intercept	1.046113	0.097907	10.6848	< 2.2e-16 ***
		Extinction slope	-0.228814	0.010881	-21.0294	< 2.2e-16 ***
		Colonisation intercept	-1.492317	0.095868	-15.5663	< 2.2e-16 ***
		Colonisation slope	0.039762	0.010643	3.7361	0.0001869***
		Negative Log Likelihood				58925.37
iii	Available species pool					
	Cold adapted					
		Extinction intercept	0.087176	0.127573	0.6833	0.4944
		Extinction slope	-0.285144	0.027464	-10.3823	< 2.2e-16 ***
		Colonisation intercept	-1.473740	0.120805	-12.1994	< 2.2e-16 ***
		Colonisation slope	0.127181	0.026010	4.8896	1.01e-06 ***
		Negative Log Likelihood				130969.8
	Cold tolerant					
		Extinction intercept	0.888782	0.139606	6.3664	1.936e-10 ***
		Extinction slope	-0.438050	0.029784	-14.7077	< 2.2e-16 ***

	Colonisation intercept	-1.739787	0.150634	-11.5498	< 2.2e-16 ***
	Colonisation slope	0.181778	0.032176	5.6495	1.609e-08 ***
	Negative Log Likelihood				127252.9
Warm tolerant					
	Extinction intercept	0.714154	0.104074	6.862	6.792e-12 ***
	Extinction slope	-0.416691	0.022447	-18.563	< 2.2e-16 ***
	Colonisation intercept	-2.160712	0.120052	-17.998	< 2.2e-16 ***
	Colonisation slope	0.261401	0.025974	10.064	< 2.2e-16 ***
	Negative Log Likelihood				112051.6
Warm adapted					
	Extinction intercept	0.60137	0.10187	5.9031	3.566e-09 ***
	Extinction slope	-0.38664	0.02436	-15.8723	< 2.2e-16 ***
	Colonisation intercept	-1.91675	0.10399	-18.4324	< 2.2e-16 ***
	Colonisation slope	0.18691	0.02487	7.5156	5.665e-14 ***
	Negative Log Likelihood				59060.71

Table 7: R packages used (including the version and citation):

**Package**	**Version**	**Citation**
tidyverse	2.0.0	Wickham H et al. (2019).*Welcome to the tidyverse. Journal of Open Source Software*, 4(43), 1686. doi: 10.21105/joss.01686
ggplot2	3.5.1	Wickham H (2016). *ggplot2: Elegant Graphics for Data Analysis.* Springer-Verlag New York. ISBN 978-3-319-24277-4. https://ggplot2.tidyverse.org
bbmle	1.0.25.1	Bolker B, R Development Core Team (2023). *bbmle: Tools for General Maximum Likelihood Estimation.* R package version 1.0.25.1. https://CRAN.R-project.org/package=bbmle
island	0.2.10	Ontiveros VJ, Capitan JA, Arthur R, Casamayor EO, Alonso D (2019). *Colonization and Extinction Rates estimated from Temporal Dynamics of Ecological Communities: The island R Package. Methods in Ecology and Evolution*, 10(7), 1108–1117. https://doi.org/10.1111/2041-210X.13176
car	3.1.3	Fox J, Weisberg S (2019). *An R Companion to Applied Regression*, Third edition. Sage, Thousand Oaks, CA. https://www.john-fox.ca/Companion/
sf	1.0.20	Pebesma E, Bivand R (2023). *Spatial Data Science: With applications in R.* Chapman and Hall/CRC. doi: 10.1201/9780429459016, https://r-spatial.org/book/
iNEXT	3.0.1	Hsieh T, Ma K, Chao A (2024). *iNEXT: Interpolation and Extrapolation for Species Diversity.* R package version 3.0.1. http://chao.stat.nthu.edu.tw/wordpress/software_download/
lme4	1.1.36	Bates D, Mächler M, Bolker B, Walker S (2015). "*Fitting Linear Mixed-Effects Models Using lme4.*" Journal of Statistical Software, 67(1), 1-48. doi:10.18637/jss.v067.i01\n<https://doi.org/10.18637/jss.v067.i01

Table 8: Moth families included as ‘macromoths’

Hepialidae
Cossidae
Limacodidae
Saturniidae
Sphingidae
Lasiocampidae
Drepanidae
Geometridae
Notodontidae
Noctuidae
Erebidae
Nolidae
