# Supplementary material for: Recent community warming of moths in Finland is driven by extinction in the north and colonisation in the south
Source: Nat Commun. 2025 Aug 12;16:7063. doi: 10.1038/s41467-025-62216-9 (PMC12344031; doi:10.1038/s41467-025-62216-9)
Supplement: Supplementary file 2 — Reporting Summary [file 41467_2025_62216_MOESM2_ESM.pdf]

nature portfolio

Corresponding author(s): Emilie Ellis
NCOMMS-24-51189-T

Last updated by author(s): 29/04/2025

Reporting Summary

Nature Portfolio wishes to improve the reproducibility of the work that we publish. This form provides structure for consistency and transparency in reporting. For further information on Nature Portfolio policies, see our Editorial Policies and the Editorial Policy Checklist.

Statistics

For all statistical analyses, confirm that the following items are present in the figure legend, table legend, main text, or Methods section.

n/a	Confirmed	
☐	☒	The exact sample size (*n*) for each experimental group/condition, given as a discrete number and unit of measurement
☐	☒	A statement on whether measurements were taken from distinct samples or whether the same sample was measured repeatedly
☐	☒	The statistical test(s) used AND whether they are one- or two-sided<br>*Only common tests should be described solely by name; describe more complex techniques in the Methods section.*
☐	☒	A description of all covariates tested
☐	☒	A description of any assumptions or corrections, such as tests of normality and adjustment for multiple comparisons
☐	☒	A full description of the statistical parameters including central tendency (e.g. means) or other basic estimates (e.g. regression coefficient) AND variation (e.g. standard deviation) or associated estimates of uncertainty (e.g. confidence intervals)
☐	☒	For null hypothesis testing, the test statistic (e.g. *F*, *t*, *r*) with confidence intervals, effect sizes, degrees of freedom and *P* value noted<br>*Give P values as exact values whenever suitable.*
☒	☐	For Bayesian analysis, information on the choice of priors and Markov chain Monte Carlo settings
☒	☐	For hierarchical and complex designs, identification of the appropriate level for tests and full reporting of outcomes
☒	☐	Estimates of effect sizes (e.g. Cohen's *d*, Pearson's *r*), indicating how they were calculated

Our web collection on statistics for biologists contains articles on many of the points above.

Software and code

Policy information about availability of computer code

Data collection	Moth data were obtained from the Finnish National Moth Monitoring Scheme. All data was opened as CSV files in Excel, but all editing was done using R (version 4.4.3) and R studio (2023.12.0+369).
Data analysis	Data analysis was done with R (version 4.4.3) and R studio (2023.12.0+369).<br>The packages we used are tidyverse (version 2.0.0), ggplot2 (version 3.5.1), bbmle (version 1.0.25.1), island (version 0.2.10), car (version 3.1.3), sf (version 1.0.20), lme4 (version 1.1.36) and iNEXT (version 3.0.1). See Supplementary Table 7.

For manuscripts utilizing custom algorithms or software that are central to the research but not yet described in published literature, software must be made available to editors and reviewers. We strongly encourage code deposition in a community repository (e.g. GitHub). See the Nature Portfolio guidelines for submitting code & software for further information.

Data

Policy information about availability of data

All manuscripts must include a data availability statement. This statement should provide the following information, where applicable:

- Accession codes, unique identifiers, or web links for publicly available datasets
- A description of any restrictions on data availability
- For clinical datasets or third party data, please ensure that the statement adheres to our policy

The raw moth data used in this study is openly available from Finnish Environment Institute, Finnish National Moth Monitoring, Biodiversity Information Facility at (https://laji.fi/en/observation/list?collectionId=HR.4511). All other data, including raw species temperature niche data and cleaned moth data used in this study are available in a Dryad database: https://doi.org/10.5061/dryad.qbzkh18s7

Research involving human participants, their data, or biological material

Policy information about studies with human participants or human data. See also policy information about sex, gender (identity/presentation), and sexual orientation and race, ethnicity and racism.

Reporting on sex and gender	n/a
Reporting on race, ethnicity, or other socially relevant groupings	n/a
Population characteristics	n/a
Recruitment	n/a
Ethics oversight	n/a

Note that full information on the approval of the study protocol must also be provided in the manuscript.

Field-specific reporting

Please select the one below that is the best fit for your research. If you are not sure, read the appropriate sections before making your selection.

☐ Life sciences ☐ Behavioural & social sciences ☒ Ecological, evolutionary & environmental sciences

For a reference copy of the document with all sections, see nature.com/documents/nr-reporting-summary-flat.pdf

Ecological, evolutionary & environmental sciences study design

All studies must disclose on these points even when the disclosure is negative.

Study description

This study examines changes in moth communities in Finland over a 30-year period (1993–2022) are responding to climate change, particularly warming trends. The research uses data from the Finnish National Moth Monitoring Scheme, comprising 224,364 individual observations across 661 species collected using light traps at multiple sites spanning a 1,200 km latitudinal gradient.

The analysis focuses on species' thermal affinities, quantified using a Species Temperature Index (STI), and characterises each site by a Community Temperature Index (CTI), which is the weighted average of STI by species abundance. Community dynamics were analysed using a stochastic colonisation-extinction model that assumes neutrality among species and estimates how community turnover rates are influenced by environmental and biological variables.

We used maximum likelihood approach to fit community level colonisation and extinction rates to our covariates: abiotic (e.g., latitude) and biotic (e.g., species pools and abundance). We then then did this analysis by grouping the community into 4 distinct thermal affinities, we then fit these covariates to examine the interactions between the groups

The experimental units are the sampling sites across Finland. Each site represents a unit where moth communities are monitored. There are 62 sites included in the final analysis, each with at least 10 years of data to ensure reliability in detecting temporal changes. The study includes multiple temporal replicates (years) for each site, capturing inter-annual variation in moth community composition and dynamics.

This comprehensive design allows for a robust analysis of how moth communities are shifting in response to climate change along a latitudinal gradient, with a focus on understanding community thermophilisation processes and associated colonisation-extinction dynamics.

Research sample

The research uses data from the Finnish National Moth Monitoring Scheme, 224,364 individual observations across 661 species

collected using light traps at multiple sites spanning a 1,200 km latitudinal gradient. We focused on “Macrolepidoptera”, since “Microlepidoptera” had been inconsistently scored from the samples. These data are also filtered for obvious sources of errors. Specifically, i) to confine our data to sites for which temporal change can be reliably established, we chose sites with at least 10 years of data; and ii) to derive a comparable baseline between sites, we excluded any sites that started data collection later than 2005.

Sampling strategy	The experimental units are the sampling sites across Finland. Each site represents a unit where moth communities are monitored. There are 62 sites included in the final analysis, each with at least 10 years of data to ensure reliability in detecting temporal changes. The study includes multiple temporal replicates (years) for each site, capturing inter-annual variation in moth community composition and dynamics.
Data collection	Moth communities were sampled using light traps (model ‘Jalas’, with 160W mixed light or 125W Hg vapour bulbs). Light traps were emptied weekly between early spring and late autumn yearly between 1993-2022 and samples were identified to the species level by volunteer experts.
Timing and spatial scale	Aggregated by year, our sites year data was between 1993-2022, with a minimum of 10 years of data at each site. Spatial, our sites captured a 1,200 km latitudinal gradient in Finland.
Data exclusions	In the current study, we focused on macro moths, since micro moths had been inconsistently scored from the samples. To ensure a robust and unbiased dataset for our analysis, we filtered the data for obvious sources of errors. Specifically, i) to confine our data to sites for which temporal change can be reliably established, we chose sites with at least 10 years of data; and ii) to derive a comparable baseline between sites, we excluded any sites that started data collection later than 2005. These filtering processes resulted in 728 species in 62 sites.<br>We gathered data on the species temperature index of 658 species from the 728 species in the filtered dataset.
Reproducibility	All the dataset cleaning and data analysis can be found in Dryad, to reproduce this work.
Randomization	This is not relevant here as this was an existing dataset. Our intense cleaning of the data ensured the sites are comparable .
Blinding	Our datasets are ecological monitoring data so this was not relvent .

Did the study involve field work? ☐ Yes ☒ No

Reporting for specific materials, systems and methods

We require information from authors about some types of materials, experimental systems and methods used in many studies. Here, indicate whether each material, system or method listed is relevant to your study. If you are not sure if a list item applies to your research, read the appropriate section before selecting a response.

Materials & experimental systems

n/a	Involved in the study	
☒	☐	Antibodies
☒	☐	Eukaryotic cell lines
☒	☐	Palaeontology and archaeology
☒	☐	Animals and other organisms
☒	☐	Clinical data
☒	☐	Dual use research of concern
☒	☐	Plants

Methods

n/a	Involved in the study	
☒	☐	ChIP-seq
☒	☐	Flow cytometry
☒	☐	MRI-based neuroimaging

Plants

Seed stocks	N/A
Novel plant genotypes	N/A
Authentication	N/A
